# Supplementary material for: Ornidazole Transfer into Colostrum and Assessment of Exposure Risk for Breastfeeding Infant: A Population Pharmacokinetic Analysis
Source: Pharmaceutics. 2023 Oct 24;15(11):2524. doi: 10.3390/pharmaceutics15112524 (PMC10675695; doi:10.3390/pharmaceutics15112524)
Supplement: Supplementary file 1 [file pharmaceutics-15-02524-s001.zip › pharmaceutics-2594452-supplementary.pdf]

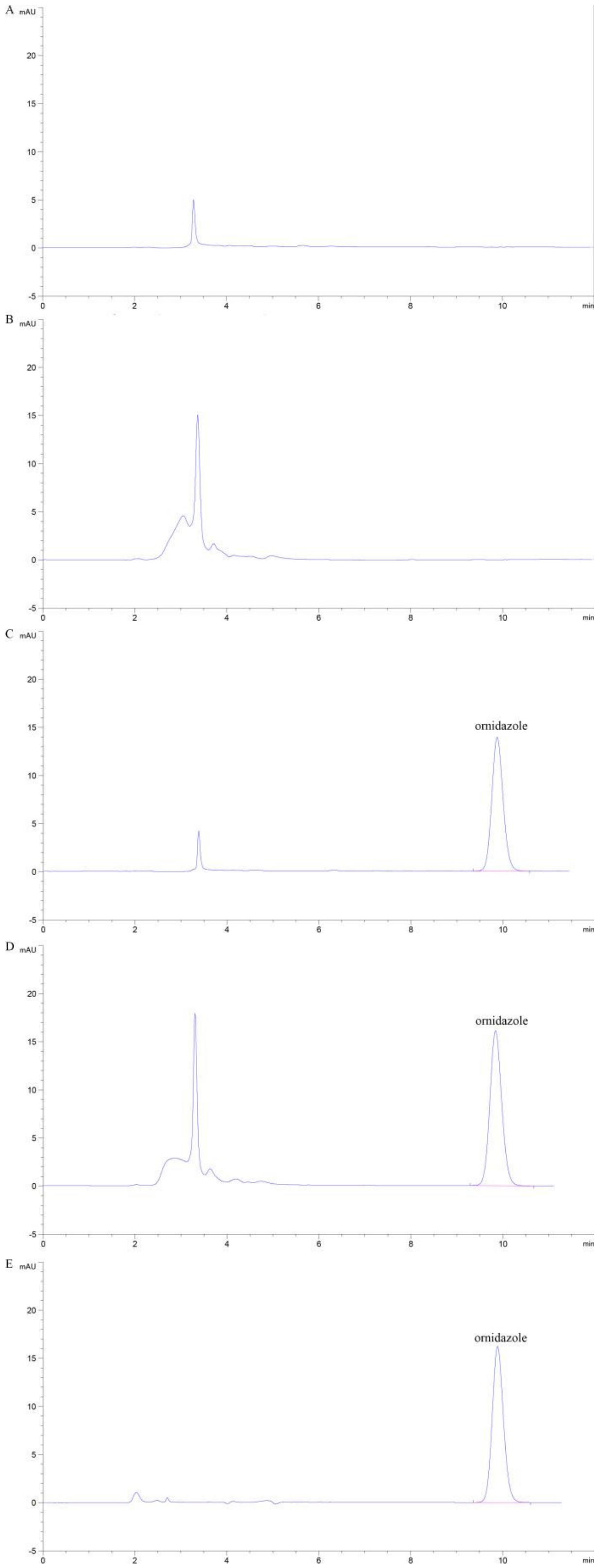

Figure S1 Typical chromatograms of (A) blank plasma, (B) blank milk, (C) blank plasma spiked with ornidazole, (D) blank milk spiked with ornidazole, and (E) aqueous solution with ornidazole.

Table S1 Candidate models to describe the relationship between plasma and milk concentration.

	Model description	Exponent (θ)	OFV	AIC	BIC	ΔOFV	Number of parameters
	Base model	-	603.18	617.18	640.61	-	7
ModelⅠ:	$MPRcon \cdot (\frac{PST}{median})^{\theta}$	estimated	565.06	581.06	607.84	-38.12	8
ModelⅡ:	$MPRcon \cdot (\frac{PST}{median})^{\theta}$	0.75	573.91	587.91	611.34	-29.27	7
ModelⅢ:	$MPRcon \cdot (\frac{PST}{median})^{\theta}$	$\theta_0 + \frac{\theta_{max} \cdot PST^{\gamma}}{\theta_{50}^{\gamma} + PST^{\gamma}}$	562.52	584.52	621.33	-40.66	11
ModelⅣ:	$MPRcon \cdot (1 + \frac{E_{max} \cdot PST^{\gamma}}{{PST_{50}}^{\gamma} + PST^{\gamma}})$	-	564.48	584.48	617.95	-38.70	10

OFV, objective function value; AIC, Akaike information criterion; BIC, Bayesian information criterion; MPRcon, milk-to-plasma concentration ratio; PST, postpartum sampling time; θ, the allometric exponent; θ_0, the exponent at a theoretical PST of zero; θ_{max}, the maximum change in the exponent; θ_{50}, the time at which the change is half of the maximum value; γ, the Hill coefficient; E_{max}, postpartum time-dependent maximum effect; PST_{50}, the PST when a 50% increase in E_{max} is achieved.

Table S2 Simulated AUC_{24} in plasma and breast milk over the first four days postpartum.

Parameter		Mean	SD	Median	IQR (Q1–Q3)
Day 1	AUC_{plasma}	474.16	34.42	474.53	452.25-497.43
	AUC_{milk}	106.33	67.69	87.27	61.45-133.15
Day 2	AUC_{plasma}	437.17	61.33	434.89	396.26-477.17
	AUC_{milk}	261.63	170.51	214.82	148.22-327.94
Day 3	AUC_{plasma}	145.58	44.40	140.93	114.63-172.48
	AUC_{milk}	153.32	110.96	122.95	81.39-189.77
Day 4	AUC_{plasma}	49.39	23.26	45.31	32.87-61.89
	AUC_{milk}	77.51	64.76	59.07	36.73-95.10

AUC_{24}, the area under the concentration-time curve over 24 hours; SD, standard deviation; IQR, interquartile range; Q1-Q3, the first to the third quartiles; AUC_{plasma}, AUC_{24} in plasma; AUC_{milk}, AUC_{24} in breast milk.
